# Psychiatric symptoms in Long-COVID patients – how really affect the quality of life?

**DOI:** 10.1192/j.eurpsy.2025.1262

**Published:** 2025-08-26

**Authors:** O. A. Caliman-Sturdza, S. Cristina

**Affiliations:** 1 Faculty of Medicine and Biological Science, Stefan cel Mare University of Suceava; 2 Infectious Diseases, Emergency Clinical County Hospital New St John Suceava, Suceava; 3 Infectious Diseases, Grigore T.Popa University Iasi, Iasi, Romania

## Abstract

**Introduction:**

Prolonged symptoms of SARS COV2 infection known as Long COVID, are defined as symptoms persisting for more than four weeks after the initial diagnosis of COVID-19. Psychiatric symptoms such as anxiety, depression, cognitive deficits, fatigue, qualities poor sleep and somatic symptoms are common in patients with long-term COVID and could last weeks, even months, after recovery.

**Objectives:**

In this study, we tried to identify the psychiatric symptoms that can start after infection with SARS COV2, what could be the risk factors for the onset of these symptoms and how the quality of life is affected in these patients.

**Methods:**

The study included 116 patients diagnosed with COVID-19 in the Suceava County Clinical Hospital, who were monitored for 12 months by applying standardized questionnaires for depression (Patient Health Questionnaire-9), anxiety (General Anxiety Disorder-7) and cognitive disorders. (Montreal Cognitive Assessment), patients being periodically evaluated clinically, biologically, neurologically and psychologically.

**Results:**

A percentage of 26.9% of patients presented the following psychiatric symptoms more than 4 weeks after the diagnosis of SARS COV2 infection: anxiety (6%), sleep disorders (7.7%) manifested by insomnia or hypersomnia, lack of concentration and attention (10.3%), memory disorders (8.6%), depression (4.3%). The most frequent psychiatric manifestations were cognitive disorders (10.3%) expressed by deficits in attention and memory, followed by sleep disorders (7.7%). Women presented a higher percentage of psychiatric manifestations manifested by anxiety and depression, but we did not find a correlation between the age of the patients and the mental manifestations after COVID-19. The 2 patients with depression in the antecedents, presented an exacerbation of mental symptoms post COVID. Depression and anxiety were more frequent in patients who had prolonged hospitalizations or moderate and severe forms of the disease. In 12 patients who presented cognitive disorders, increased values of C-reactive protein and lactic dehydrogenase were found during SARS COV2 infection. The use of questionnaires showed an important impact on the quality of life, especially in patients diagnosed with depression, anxiety and insufficient sleep.

**Conclusions:**

Recognition and treatment of psychiatric symptoms that started after SARS COV2 infection should be included in the management of the patient with chronic COVID. The use of screening questionnaires for depression, anxiety and cognitive disorders must be implemented in the current practice of post-COVID-19 monitoring. The collaboration between infectious disease specialist, psychiatrist and psychologist can be the key to success in the treatment of psychiatric disorders in patients with long-term COVID.

**Disclosure of Interest:**

None Declared

